# Heterogeneity in intracranial relapses after complete resection of lung adenocarcinoma: Distinct features of brain‐only relapse versus synchronous extracranial relapse

**DOI:** 10.1002/cam4.5961

**Published:** 2023-04-16

**Authors:** Fei Xu, Junling Li, Puyuan Xing, Yutao Liu, Yan Wang

**Affiliations:** ^1^ Department of Medical Oncology, National Cancer Center/National Clinical Research Center for Cancer/Cancer Hospital Chinese Academy of Medical Sciences and Peking Union Medical College Beijing China

**Keywords:** brain metastases, complete resection, *EGFR*, non‐small cell lung cancer

## Abstract

**Background:**

Patients with brain oligometastases have better prognosis than those with synchronous extracranial metastases in non‐small cell lung cancer (NSCLC). However, studies focusing on intracranial‐only recurrence after curative surgery remained scarce. This study aimed to explore distinct features of patients with exclusive brain relapse after resection of lung adenocarcinoma.

**Methods:**

Records were retrospectively reviewed of 2809 patients who had complete resection and pathologically confirmed stage IB‐IIIA NSCLC in our hospital from October 2012 to September 2019. Patients were enrolled if they were adenocarcinoma and developed intracranial recurrence thereafter. They were divided into two groups depending on whether they had synchronous extracranial metastases. Clinical and pathological features of patients enrolled were collected and compared between groups.

**Results:**

Ninety‐seven lung adenocarcinoma patients with intracranial recurrences were enrolled. The median follow‐up time was 40 months. Fifty patients (51.5%) had brain oligometastases and 47 patients had synchronous extracranial metastases (ECM). Multivariate logistic regression suggested *EGFR*‐sensitive mutation and male sex were positively correlated to brain‐only recurrence (OR = 2.59, 95%CI 1.04–6.84 and OR = 2.58, 95% CI 1.05–6.75), while higher clinical stage was associated with synchronous ECM (stage II (OR = 0.33, 95%CI 0.09–1.14) or stage IIIA (OR = 0.54, 95%CI 0.20–1.38) versus stage I). No other pathological feature (lymphovascular invasion, visceral pleural invasion, low tumor differentiation, etc.) or adjuvant chemotherapy was associated with intracranial‐only relapse after complete resection of primary tumor.

**Conclusion:**

Among patients with brain relapse after resection of lung adenocarcinoma, patients with EGFR mutations might have intracranial relapse only without synchronous extracranial metastases. Further prospective studies are warranted to verify this.

## Graphical abstract

Among patients with brain relapse after complete resection of lung adenocarcinoma, patients with EGFR mutations might have intracranial relapse only without synchronous extracranial metastases. Further prospective studies are warranted to verify this.

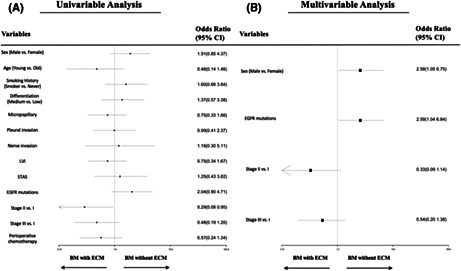

## 1 INTRODUCTION

Brain metastases has long been a vital challenge for cancer treatment.^1^ Though the overall prognosis remained poor for malignancies with brain metastases, oligometastases without extracranial lesions could have longer survival,^2^, ^3^ due to effective local therapies^4^, ^5^, ^6^ and more importantly, their distinct tumor biology.^7^, ^8^ Since blood–brain barrier (BBB) has set great challenges for cancer cells through metastases cascade biologically,^9^ it is of great interest why some patients undergone brain oligometastases without extracranial sites, which was less challenged route for cancer cells. This is of great clinical concern since the monitoring of the brain remains controversial for early‐stage cancer^10^ and is less frequently considered than body computed tomography (CT) scans for patients after complete resection of primary tumor.

Lung cancer, holding the highest cancer mortality in China and around the world,^11^, ^12^ is the most common type of malignancy to develop brain metastases.^13^, ^14^, ^15^ About one‐third of non‐small cell lung cancer (NSCLC) patients would develop intracranial metastases during disease course. At initial diagnosis, 57% of lung cancer patients already had distant metastasis, among whom 26%–32% were diagnosed with brain metastasis.^16^, ^17^ Moreover, early‐stage NSCLC patients also bear the risk for brain metastasis even after radical surgery or radiotherapy, at a rate of nearly 10%.^18^ However, treatment for brain metastases have been more directed on advanced NSCLC with existed brain metastases,^19^, ^20^, ^21^, ^22^ while it has not been widely recognized regarding prophylaxis of brain metastases in high‐risk patients,^23^ though there have been several clinical trials revealing benefit with prophylactic cranial irradiation^24^, ^25^

Lung adenocarcinoma (LUAD) has been regarded as a high‐risk population to develop brain metastases among NSCLC after complete resection of primary tumor, holding 11% of patients at risk of brain metastases, higher than its counterpart, squamous cell lung carcinoma (6%).^18^ Previous studies have hinted several factors related to brain recurrences after primary tumor resection.^26^, ^27^ However, more detailed characteristics concerning resected tumor had not been elucidated for their correlation with brain oligometastases.

It Is an unmet need for oncologists to distinguish patients with brain oligometastases from those with synchronous extracranial metastases (ECM) at initial relapse after complete resection. Because (1) brain‐only recurrences may have distinct tumor biology and could potentially cured by local therapy, (2) there has been no approved prophylactic treatment toward brain recurrence in non‐small cell lung cancer. This study focused on intracranial relapsed LUAD patients with or without synchronous ECM, aimed to find distinct features of brain oligometastases, hoping to add more information on monitoring after surgery for early‐stage NSCLC.

## 2 METHODS

### 2.1 Patients and follow‐up

We retrospectively reviewed the medical records of 2809 patients who had undergone radical lobectomy of lung tumor from October 2012 to September 2019, in order to find the patients who had intracranial relapsed disease after complete resection. The inclusion criteria were: (1) Patients should have no distant metastases before surgery in following imaging examinations: CT scans for chest and abdomen, plus MRI for brain; or PET‐CT scan of the whole body. (2) The pathology after surgery should be lung adenocarcinoma, with pathologically assured stage IB‐IIIA. (3) Patients should have relapsed disease during follow‐up after surgery. (4) Patients should have at least one site of brain metastases during follow‐up after surgery. The exclusion criteria were: (1) Patients had non‐complete resection of the primary lung cancer, including R1/R2 resection or substandard lymph node dissection. (2) Patients had other primary malignancies. (3) The intracranial lesions were undefined on imaging examination. (4) unknown actionable mutation status (including *EGFR* mutation, *KRAS* mutation and *ALK* rearrangement).

For patients who met the criteria, clinical and pathological information was retrospectively collected. Clinical characteristics included gender, age at diagnosis, smoking history, and date of complete resection. Pathological characteristics included histologic subtypes, tumor differentiation, micropapillary, pleural invasion status, nerve invasion, lymphovascular invasion (LVI), spread through air space (STAS), driver gene mutations (see “Molecular testing” below) and TNM stage. Therapeutic information was also collected with regard to neoadjuvant therapy and adjuvant therapy. The follow‐up of patients was routinely arranged as following: CT scans for chest and abdomen every 3 months within 2 years after surgery, then every 6 months within 5 years after surgery, and yearly examination 5 years after surgery; MRI for brain when patients had symptoms or routinely every 6 months. Contrast mediums were required unless patient was intolerant to it. The last follow‐up date was on Aug 10th, 2021. Disease‐free survival (DFS) was calculated as the time between the date of complete resection and the date of disease relapse, which was acquired from follow‐up clinic visits

This study was approved by Ethics Committee of National Cancer Center /National Clinical Research Center for Cancer/Cancer Hospital, Chinese Academy of Medical Sciences and Peking Union Medical College in accordance with the declaration of Helsinki protocol. Written informed consent was waived because this was a retrospective study and neither the implement nor the outcome of this study would do harm to patients involved in this study.

### 2.2 Definition of brain‐only metastases and brain‐with metastases

Patients who met the criteria for this study were divided into two groups depending on whether synchronous ECM existed within 6 months of diagnosis of brain metastases (the time of 6 months was set for a balance of imaging frequency and treatment impact). Patients were defined as the BM‐only group if they only had brain metastasis as the first relapsed site, and did not have extracranial metastasis within the next 6 months; other patients with ECM were defined as BM‐with group.

### 2.3 Molecular testing of resected tumor tissues

*EGFR* and *KRAS* mutations were tested by PCR, and *ALK* mutations were tested by immunohistochemistry of tumor tissue, which was recorded in the digital medical records in our hospital. Other genes were not tested since panel‐based next‐generation sequencing was not used as a clinical routine in earlier years.

### 2.4 Statistical analysis

The Kaplan–Meier analysis was used to generate the survival curves and the Log‐Rank test was employed to compare the difference among the curves. Univariate and multivariate Logistic regression model was applied to identify risk factors of intracranial relapse with or without ECM. A *p* value <0.05 was considered statistically significant and all statistical tests were two‐sided. All statistical analyses were conducted using R software (version 4.0.3).

## 3 RESULTS

### 3.1 Baseline characteristics of patients

**FIGURE 1:**
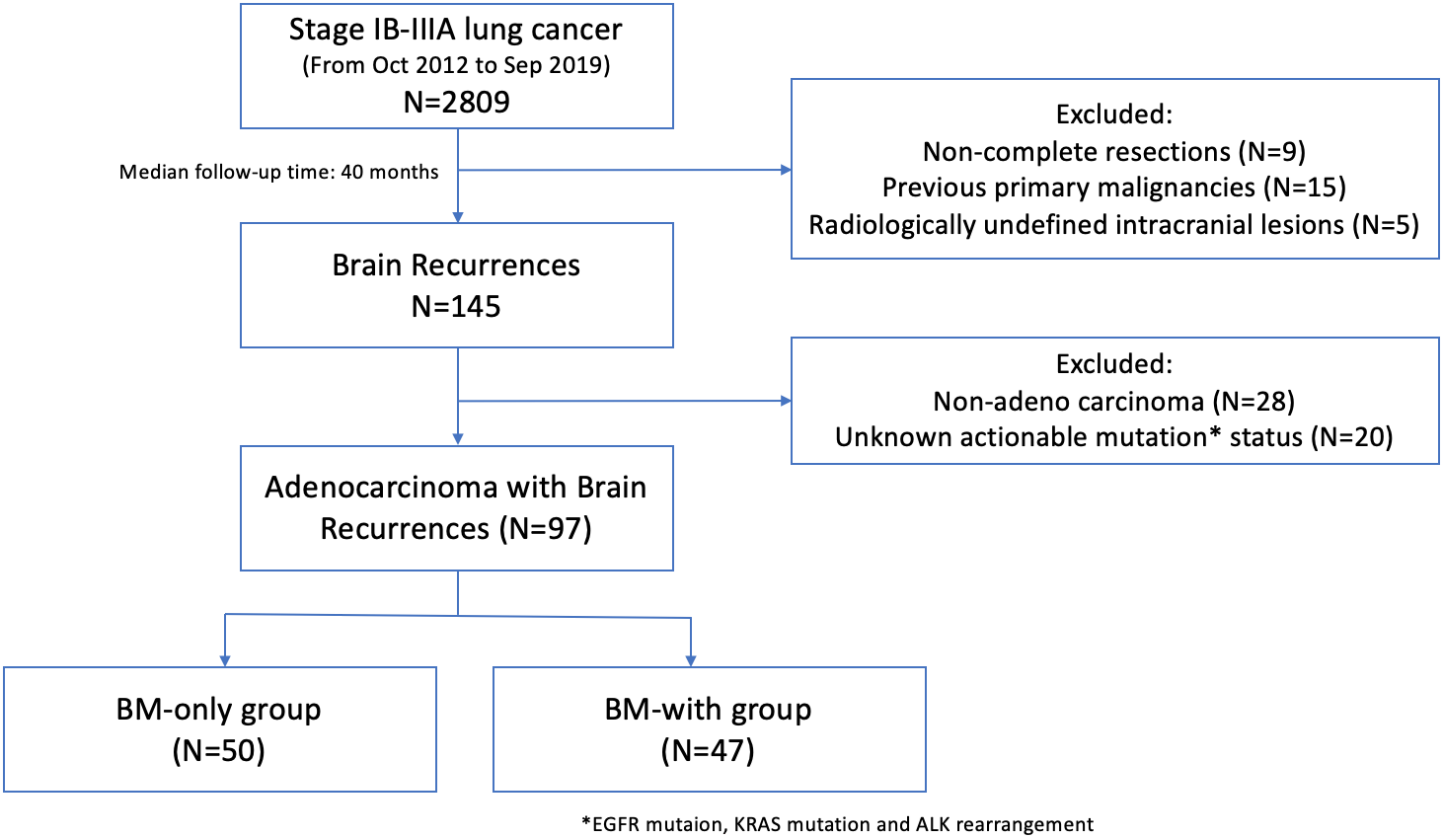
Flow‐chart of the study.

After reviewing the medical records of 2809 patients who underwent radical mastectomy of lung tumors with pathologically confirmed stage IB to IIIA in our center from October 2012 to September 2019, 145 (5.2%) patients had been recorded to have brain recurrences. After exclusion of non‐adeno carcinoma patients and patients with unknown actionable mutation status, 97 lung adenocarcinoma patients with intracranial relapse with or without ECM were included for analysis. Detailed flow‐chart was demonstrated in Figure 1. The median follow‐up time was 40 months. Baseline clinical and pathological characteristics were included in Table 1, with detailed information in Table S1.

**TABLE 1:** Baseline clinical and pathological characteristics of intracranial relapsed patients.

		All (*N* = 97)
Age at diagnosis	<65 yrs	82 (85%)
	≥65 yrs	15 (15%)
Gender	Male	43 (44%)
	Female	54 (56%)
Smoking history	Never	65 (67%)
	Smoker	32 (33%)
Differentiation	Low	69 (71%)
	Medium	28 (29%)
Micropapillary	Yes	50 (52%)
	No	47 (48%)
TNM stage	IB	32 (33%)
	IIA‐B	17 (17%)
	IIIA	48 (50%)
Pleural invasion	Yes	68 (70%)
	No	29 (30%)
Nerve invasion	Yes	9 (9%)
	No	88 (91%)
Lymphovascular invasion (LVI)	Yes	46 (47%)
	No	51 (52%)
Tumor spread through air space (STAS)	Yes	16 (16%)
	No	81 (84%)
Driver mutation status	EGFR	58 (60%)
	KRAS	14 (14%)
	ALK fusions	6 (6%)
Perioperative chemotherapy	Yes	64 (66%)
	No	33 (34%)

### 3.2 Disease‐free survival of patients with intracranial relapse with or without ECM

**FIGURE 2:**
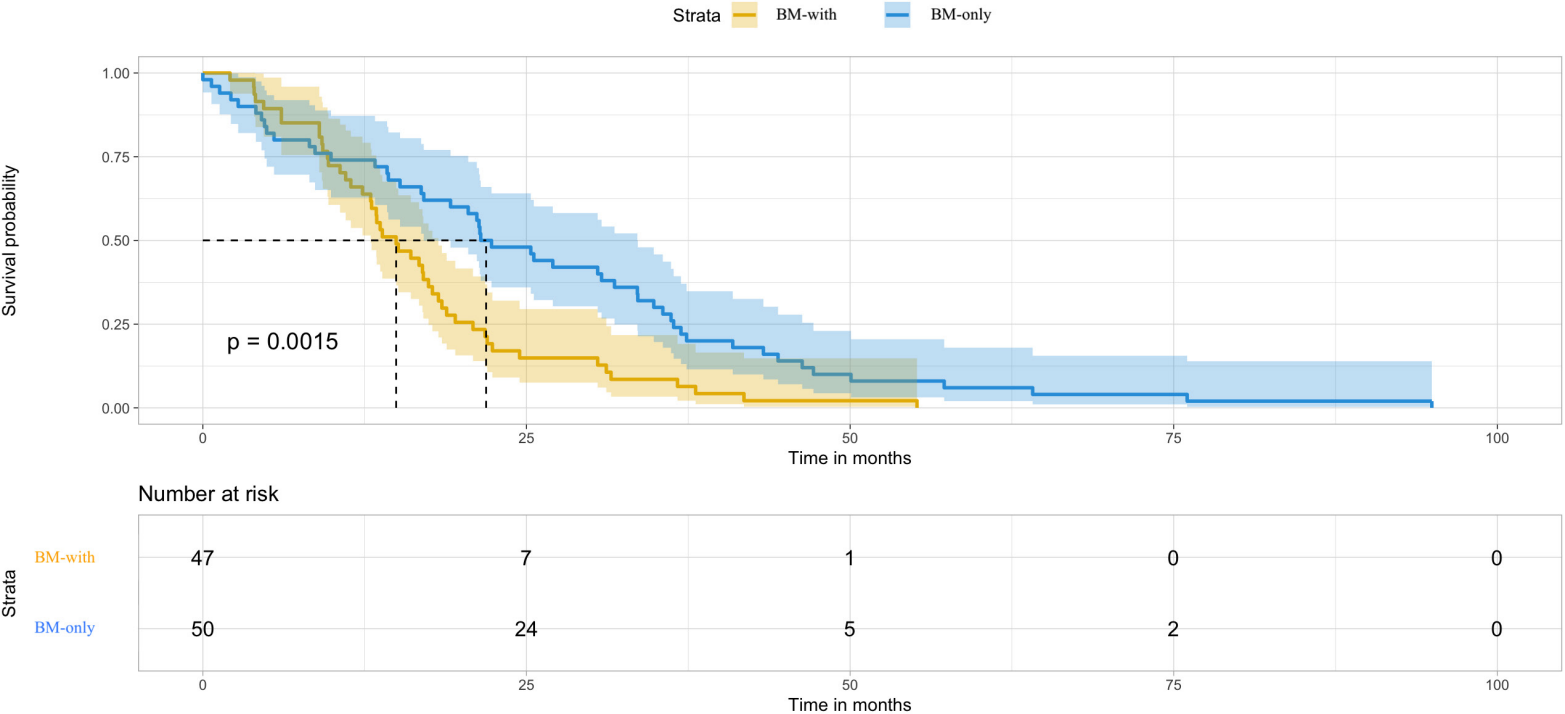
Disease‐free survival (DFS) among intracranial relapsed patients with or without extracranial metastases. Patients were divided into two groups depending on whether harboring synchronous ECM: patients were defined as the BM‐only group if they only had brain metastasis as the first relapsed site while other patients were defined as BM‐with group. BM‐only group had longer than BM‐with group (22.3 vs. 15.1 months, *p* = 0.0015).

Fifty patients (51.5%) had brain oligometastases (BM‐only group) and 47 patients had synchronous ECM (BM‐with group). We found that BM‐only group had significantly longer DFS than BM‐with group (22.3 vs. 15.1 months, *p* = 0.0015) (Figure 2).

### 3.3 Radiographic data of intracranial relapse lesions with or without ECM

The radiographic features of brain metastases lesions were collected and analyzed. The radiographic data were demonstrated in Table S2, including number of brain metastases, site of brain metastases (i.e., cerebrum or cerebellum), location of brain metastases (i.e., right or left sided) and the largest diameter of brain lesions. More than half of brain metastases appeared as single lesion, while there was no significant difference between BM‐only group and BM‐with group (*p* = 0.064). Cerebrum was more commonly involved than cerebellum, while there was no significant difference between groups (*p* = 0.275). There was also no difference between groups with regard to brain metastases location (*p* = 0.216) or lesion size (*p* = 0.246).

### 3.4 Characteristics related to intracranial relapse with or without ECM

**FIGURE 3:**
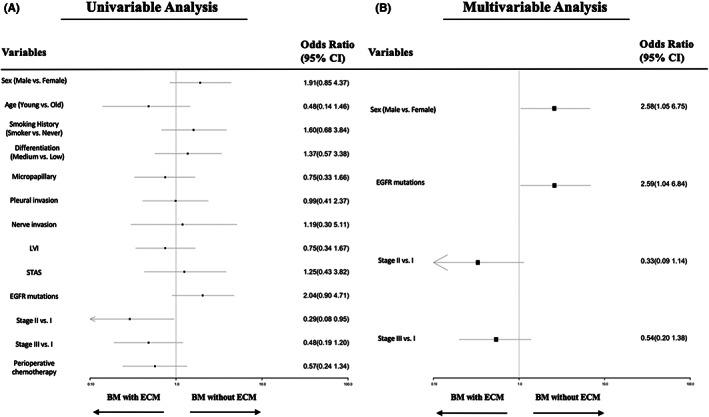
Univariate (A) and multivariate (B) analysis for the risk of intracranial metastases without extracranial metastases. The odds ratio of intracranial metastases‐only was estimated by logistic regression model. The odds ratio for each variable was counted compared to their counterpart as the reference value, for example: male patients versus female patients, OR = 1.91(0.85 4.37) in the univariate analysis and OR = 2.58(1.05 6.75) in the multivariate analysis.

Next, we managed to explore whether there were different clinical and pathological features between patients with intracranial relapse with or without ECM. We managed to find differences between BM‐only group and BM‐with group in terms of gender, age at diagnosis, smoking history, histologic subtypes, tumor differentiation, micropapillary, pleural invasion status, nerve invasion, LVI, STAS, driver gene mutations and perioperative chemotherapy. The result of univariate and multivariate logistic analysis was elucidated in Figure 3A,B. Male patients tended to have brain relapse without extracranial involvement (OR = 2.58, 95% CI 1.05–6.75), while age seemed not a factor in distinguishing two groups (OR = 0.48, 95% CI 0.14–1.46). Pathological features including tumor differentiation (OR = 1.37, 95% CI 0.57–3.38), micropapillary (OR = 0.75, 95% CI 0.33–1.66), pleural invasion (OR = 0.99, 95% CI 0.41–2.37), nerve invasion (OR = 1.19, 95% CI 0.30–5.11), lymphovascular invasion (OR = 0.75, 95% CI 0.34–1.67) and STAS (OR = 1.25, 95% CI 0.43–3.82), which were regarded as risk factors for disease recurrence, failed to distinguish patients at high risk of relapse at both intracranial and extracranial sites. Perioperative chemotherapy (OR = 0.57, 95% CI 0.24–1.34) had no effect on distinguishing BM‐only patients from BM‐with patients. *EGFR*‐sensitive mutation was found to have tendency toward intracranial metastases only (OR = 2.59, 95%CI 1.04–6.84). Compared to stage IB patients, stage II (OR = 0.33, 95%CI 0.09–1.14) or stage IIIA (OR = 0.54, 95%CI 0.20–1.38) patients tended to have synchronous BM and ECM.

## 4 DISCUSSION

It has been recognized that the prognosis of cancer patients with brain metastases is affected by the status and the number of synchronous extracranial metastases(ECM),^28^, ^29^ and even the status of lymph node metastases.^30^ However, which group of patients might develop brain oligometastases remained a problem. In order to explore the distinct features of early‐stage lung adenocarcinoma patients who developed brain oligometastases, we compared brain‐only relapse (oligometastases) group with brain‐with relapse (synchronous ECM) group. In this study, about half of intracranial relapsed patients had brain as the sole site of recurrence, which is comparable to that of 43% in a previous study.^31^ Higher clinical stage is associated with synchronous ECM, which was in line with other studies.^26^, ^31^ Besides, previous studies focusing on brain metastases risk factors showed that younger age, lymphovascular invasion, micropapillary subtype and multiple lymph nodes involvement were correlated with brain metastases,^26^, ^27^, ^31^, ^32^ while primary tumor differentiation, pleural invasion, nerve invasion, lymphovascular invasion and STAS were regarded as risk factors for disease relapse.^33^, ^34^, ^35^, ^36^, ^37^ However, our results here showed that none of the clinical or pathological features mentioned above was associated with brain‐only relapse after complete resection of primary tumor for early‐stage NSCLC. This indicate that there might be different underlying mechanisms between brain oligometastases and disease recurrence after complete resection of primary tumor.

Interestingly, our results demonstrated that *EGFR*‐sensitive mutations was significantly associated with brain oligometastases. *EGFR*‐mutated NSCLC was found to be correlated with high frequency of brain metastases in many studies before.^38^, ^39^, ^40^, ^41^, ^42^, ^43^ However, inquiry on brain‐only metastases after surgery is scarce. A previous small‐sized study analyzed 28 patients with brain recurrence after curative surgery, and found a higher frequency of brain‐only pattern in *EGFR*‐mutated group (11/16) than wild‐type group (3/12).^44^ Together, these two retrospective studies both indicated the correlation of *EGFR* mutation with brain oligometastases in post‐resection setting. However, this conclusion needs further validation from perspective studies and the mechanisms behind this distinct metastatic biology remains to be investigate. One assumption is that *EGFR* mutation is an early and clonal event in tumor evolution,^45^ and tumor harboring *EGFR* mutations seemed to act in line with “attenuated progression”, described as initial oligometastases and gradually gaining metastatic capacity over time.^46^ This might have given an explanation as why *EGFR*‐mutated NSCLC patients are more inclined to have brain metastases but not bearing with decreased survival.^47^

Our findings have clinical implications. Firstly, our results indicated that closely brain MRI monitoring should be considered for *EGFR*‐mutated patients within 2 years after complete resection, even when body CT scans showed no sign of disease relapse. This is very important because brain MRIs are often prescribed when neurological symptoms occur in real‐world settings, although guidelines have recommended routine brain screening for stage II‐III NSCLC after curative treatment.^48^ Secondly, two randomized clinical trials (ADAURA and EVIDENCE) both indicated that *EGFR* targeted therapies (osimertinib/icotinib) could delay disease recurrence.^49^, ^50^ Based on our results, we are inclined to recommend drugs with higher intracranial potency (osimertinib) for adjuvant therapy, anticipating a “cure” outcome for patients with brain oligometastases which are more common in *EGFR*‐mutated patients.

There were limitations of this study. First, as a retrospective study, this study had inherent disadvantage in selection bias, that was, some patients might had relapsed but failed to revisit our hospital. Second, the study was analyzed based on data available in medical records, so that information on genetic mutations was relatively scarce. Future studies might take advantage of whole‐exon or next‐generation sequencing to get a full view of genetic variations including co‐mutations. More biological processes and biomarkers need to be investigated, both in irreversible genetic variations and reversible processes such as DNA methylation and metabolomics.

## 5 CONCLUSIONS

Among patients with brain relapse after resection of lung adenocarcinoma, patients with EGFR mutations might have intracranial relapse only without synchronous extracranial metastases. Further prospective studies are warranted to verify this.

## AUTHOR CONTRIBUTIONS

**Fei Xu:** Data curation (lead); formal analysis (lead); methodology (equal); visualization (lead); writing – original draft (lead). **Junling Li:** Resources (equal); supervision (equal); validation (equal). **Puyuan Xing:** Resources (equal); supervision (equal); validation (equal). **Yutao Liu:** Resources (equal); supervision (equal); validation (equal). **Yan Wang:** Conceptualization (lead); project administration (lead); writing – review and editing (lead).

## CONFLICT OF INTEREST STATEMENT

The authors declare that they have no competing interests.

## ETHICS STATEMENT

This study was approved by Ethics Committee of National Cancer Center /National Clinical Research Center for Cancer/Cancer Hospital, Chinese Academy of Medical Sciences and Peking Union Medical College in accordance with the declaration of Helsinki protocol. The ethical number of this study is 21/163–2834. Written informed consent was waived because this was a retrospective study and neither the implement nor the outcome of this study would do harm to patients involved in this study.

## Supporting information


Table S1.

Table S2.

## Data Availability

Data, models, or code generated or used during the study are available from the corresponding author on reasonable request.
