# Supplementary material for: Heterogeneity in intracranial relapses after complete resection of lung adenocarcinoma: Distinct features of brain‐only relapse versus synchronous extracranial relapse
Source: Cancer Med. 2023 Apr 16;12(11):12495–503. doi: 10.1002/cam4.5961 (PMC10278484; doi:10.1002/cam4.5961)
Supplement: Supplementary file 1 — Table S1. [file CAM4-12-12495-s002.docx]

|  | **Supplementary Table 1 Detailed baseline characteristics of 97 patients** | | | | | | | | | |
| --- | --- | --- | --- | --- | --- | --- | --- | --- | --- | --- |
| ID | Sex | Age | Smoking History | Surgery-Date | Pathology | Differentiation Grade | Main-subtype | T | N | Stage |
| 1 | Female | 63 | never | 2013/8/6 | adenocarcinoma | medium | acinar | 3 | 0 | II |
| 2 | Female | 65 | never | 2015/10/29 | adenocarcinoma | medium | papillary | 3 | 0 | II |
| 3 | Female | 67 | never | 2016/3/25 | adenocarcinoma | poorly | acinar | 4 | 2 | III |
| 4 | Female | 67 | never | 2018/3/20 | adenocarcinoma | poorly | acinar | 4 | 2 | III |
| 5 | Female | 43 | never | 2018/4/24 | adenocarcinoma | poorly | papillary | 3 | 2 | III |
| 6 | Female | 62 | never | 2018/12/25 | adenocarcinoma | poorly | acinar | 3 | 2 | III |
| 7 | Male | 64 | never | 2019/1/3 | adenocarcinoma | poorly | acinar | 2 | 2 | III |
| 8 | Female | 55 | never | 2019/2/22 | adenocarcinoma | poorly | solid | 2 | 2 | III |
| 9 | Male | 53 | smoked/smoking | 2019/3/18 | adenocarcinoma | poorly | solid | 2 | 2 | III |
| 10 | Male | 66 | smoked/smoking | 2019/4/8 | adenocarcinoma | medium | acinar | 2 | 2 | III |
| 11 | Female | 37 | smoked/smoking | 2019/4/16 | adenocarcinoma | poorly | acinar | 1 | 2 | III |
| 12 | Female | 36 | never | 2012/12/14 | adenocarcinoma | medium | acinar | 2 | 0 | I |
| 13 | Female | 60 | never | 2013/4/12 | adenocarcinoma | medium | papillary | 2 | 0 | I |
| 14 | Female | 67 | never | 2013/6/21 | adenocarcinoma | medium | papillary | 2 | 0 | I |
| 15 | Male | 57 | smoked/smoking | 2014/2/27 | adenocarcinoma | poorly | acinar | 2 | 0 | I |
| 16 | Female | 53 | never | 2014/9/18 | adenocarcinoma | medium | acinar | 2 | 0 | I |
| 17 | Male | 48 | smoked/smoking | 2014/10/11 | adenocarcinoma | poorly | acinar | 2 | 0 | I |
| 18 | Female | 68 | never | 2014/11/14 | adenocarcinoma | medium | acinar | 2 | 0 | I |
| 19 | Male | 67 | smoked/smoking | 2015/4/13 | adenocarcinoma | medium | acinar | 2 | 0 | I |
| 20 | Female | 63 | never | 2015/7/17 | adenocarcinoma | medium | acinar | 2 | 0 | I |
| 21 | Female | 49 | never | 2015/10/15 | adenocarcinoma | medium | acinar | 2 | 0 | I |
| 22 | Female | 64 | never | 2015/12/12 | adenocarcinoma | medium | acinar | 2 | 0 | I |
| 23 | Male | 64 | smoked/smoking | 2016/2/18 | adenocarcinoma | medium | acinar | 2 | 0 | I |
| 24 | Female | 53 | never | 2014/7/8 | adenocarcinoma | poorly | acinar | 3 | 0 | II |
| 25 | Female | 53 | never | 2016/4/7 | adenocarcinoma | poorly | solid | 2 | 2 | III |
| 26 | Male | 72 | smoked/smoking | 2017/2/10 | adenocarcinoma | poorly | papillary | 2 | 1 | II |
| 27 | Female | 51 | never | 2017/6/12 | adenocarcinoma | poorly | acinar | 1 | 1 | II |
| 28 | Male | 51 | smoked/smoking | 2017/6/13 | adenocarcinoma | poorly | solid | 1 | 2 | III |
| 29 | Female | 33 | never | 2017/7/6 | adenocarcinoma | poorly | acinar | 3 | 1 | III |
| 30 | Male | 50 | never | 2017/7/11 | adenocarcinoma | poorly | acinar | 1 | 2 | III |
| 31 | Female | 56 | never | 2017/8/1 | adenocarcinoma | poorly | acinar | 4 | 2 | III |
| 32 | Female | 53 | never | 2017/11/7 | adenocarcinoma | poorly | acinar | 3 | 2 | III |
| 33 | Male | 60 | never | 2017/11/17 | adenocarcinoma | poorly | solid | 2 | 2 | III |
| 34 | Male | 55 | never | 2017/11/28 | adenocarcinoma | poorly | acinar | 3 | 2 | III |
| 35 | Female | 43 | never | 2017/12/21 | adenocarcinoma | poorly | papillary | 2 | 2 | III |
| 36 | Male | 52 | never | 2017/12/25 | adenocarcinoma | poorly | papillary | 3 | 2 | III |
| 37 | Female | 52 | never | 2018/1/19 | adenocarcinoma | medium | acinar | 2 | 1 | II |
| 38 | Male | 47 | smoked/smoking | 2018/3/2 | adenocarcinoma | poorly | acinar | 3 | 2 | III |
| 39 | Female | 54 | never | 2018/4/9 | adenocarcinoma | poorly | acinar | 1 | 2 | III |
| 40 | Female | 51 | never | 2018/7/19 | adenocarcinoma | poorly | acinar | 2 | 2 | III |
| 41 | Female | 45 | never | 2018/8/10 | adenocarcinoma | poorly | micropapillary | 1 | 2 | III |
| 42 | Female | 47 | never | 2018/9/17 | adenocarcinoma | poorly | acinar | 2 | 1 | II |
| 43 | Female | 68 | never | 2018/12/28 | adenocarcinoma | poorly | solid | 2 | 2 | III |
| 44 | Female | 49 | never | 2019/2/22 | adenocarcinoma | poorly | solid | 1 | 1 | II |
| 45 | Female | 59 | never | 2019/2/26 | adenocarcinoma | poorly | solid | 2 | 2 | III |
| 46 | Female | 64 | never | 2019/3/4 | adenocarcinoma | medium | acinar | 1 | 2 | III |
| 47 | Male | 62 | smoked/smoking | 2019/3/22 | adenocarcinoma | medium | acinar | 2 | 2 | III |
| 48 | Female | 49 | never | 2019/4/15 | adenocarcinoma | poorly | acinar | 2 | 0 | II |
| 49 | Male | 66 | never | 2013/1/24 | adenocarcinoma | poorly | acinar | 2 | 0 | I |
| 50 | Female | 59 | never | 2014/1/14 | adenocarcinoma | medium | acinar | 2 | 0 | I |
| 51 | Female | 62 | never | 2014/12/15 | adenocarcinoma | poorly | papillary | 2 | 0 | I |
| 52 | Female | 59 | never | 2014/12/25 | adenocarcinoma | medium | acinar | 2 | 0 | I |
| 53 | Male | 59 | smoked/smoking | 2015/1/6 | adenocarcinoma | medium | acinar | 2 | 0 | I |
| 54 | Female | 46 | never | 2015/1/21 | adenocarcinoma | medium | acinar | 2 | 0 | I |
| 55 | Male | 62 | smoked/smoking | 2015/2/10 | adenocarcinoma | medium | acinar | 2 | 0 | I |
| 56 | Female | 36 | never | 2015/5/29 | adenocarcinoma | medium | acinar | 2 | 0 | I |
| 57 | Female | 45 | never | 2015/11/13 | adenocarcinoma | poorly | papillary | 2 | 0 | I |
| 58 | Female | 50 | never | 2015/11/17 | adenocarcinoma | poorly | solid | 2 | 0 | I |
| 59 | Male | 60 | smoked/smoking | 2013/10/15 | adenocarcinoma | medium | papillary | 3 | 0 | II |
| 60 | Female | 54 | never | 2017/5/16 | adenocarcinoma | poorly | acinar | 2 | 2 | III |
| 61 | Female | 64 | never | 2017/5/18 | adenocarcinoma | poorly | acinar | 2 | 2 | III |
| 62 | Male | 70 | never | 2017/8/21 | adenocarcinoma | poorly | acinar | 2 | 2 | III |
| 63 | Male | 48 | smoked/smoking | 2018/5/22 | adenocarcinoma | poorly | solid | 1 | 2 | III |
| 64 | Male | 59 | smoked/smoking | 2018/7/4 | adenocarcinoma | poorly | papillary | 1 | 2 | III |
| 65 | Male | 59 | smoked/smoking | 2018/7/12 | adenocarcinoma | medium | acinar | 2 | 1 | II |
| 66 | Male | 57 | never | 2013/4/1 | adenocarcinoma | poorly | acinar | 2 | 0 | I |
| 67 | Male | 51 | smoked/smoking | 2013/10/12 | adenocarcinoma | medium | acinar | 2 | 0 | I |
| 68 | Female | 64 | never | 2014/3/17 | adenocarcinoma | poorly | acinar | 2 | 0 | I |
| 69 | Female | 68 | never | 2014/9/18 | adenocarcinoma | poorly | acinar | 2 | 0 | I |
| 70 | Male | 68 | smoked/smoking | 2015/5/22 | adenocarcinoma | medium | acinar | 2 | 0 | I |
| 71 | Male | 75 | smoked/smoking | 2015/8/21 | adenocarcinoma | poorly | acinar | 2 | 0 | I |
| 72 | Female | 42 | never | 2013/10/11 | adenocarcinoma | poorly | solid | 1 | 1 | II |
| 73 | Male | 59 | smoked/smoking | 2013/12/25 | adenocarcinoma | poorly | poorly | 2 | 0 | II |
| 74 | Male | 52 | smoked/smoking | 2014/9/27 | adenocarcinoma | medium | acinar | 3 | 2 | III |
| 75 | Female | 62 | never | 2014/12/12 | adenocarcinoma | poorly | mucinous | 3 | 1 | III |
| 76 | Female | 48 | never | 2017/4/26 | adenocarcinoma | medium | papillary | 4 | 2 | III |
| 77 | Male | 72 | smoked/smoking | 2017/5/18 | adenocarcinoma | poorly | solid | 2 | 2 | III |
| 78 | Male | 49 | never | 2017/7/27 | adenocarcinoma | poorly | acinar | 1 | 1 | II |
| 79 | Female | 53 | never | 2017/8/16 | adenocarcinoma | poorly | solid | 1 | 2 | III |
| 80 | Female | 54 | never | 2017/8/21 | adenocarcinoma | poorly | papillary | 4 | 2 | III |
| 81 | Male | 59 | smoked/smoking | 2017/11/3 | adenocarcinoma | poorly | solid | 2 | 1 | II |
| 82 | Male | 38 | never | 2017/11/21 | adenocarcinoma | poorly | micropapillary | 2 | 1 | II |
| 83 | Male | 60 | smoked/smoking | 2018/1/29 | adenocarcinoma | poorly | acinar | 1 | 2 | III |
| 84 | Female | 53 | never | 2018/5/15 | adenocarcinoma | poorly | solid | 2 | 2 | III |
| 85 | Male | 50 | never | 2018/6/21 | adenocarcinoma | poorly | solid | 1 | 2 | III |
| 86 | Male | 57 | smoked/smoking | 2018/8/9 | adenocarcinoma | poorly | solid | 2 | 2 | III |
| 87 | Female | 38 | never | 2018/9/28 | adenocarcinoma | poorly | solid | 2 | 2 | III |
| 88 | Female | 58 | never | 2018/12/5 | adenocarcinoma | poorly | acinar | 2 | 1 | II |
| 89 | Male | 49 | smoked/smoking | 2018/12/13 | adenocarcinoma | poorly | acinar | 2 | 2 | III |
| 90 | Female | 41 | never | 2018/12/29 | adenocarcinoma | poorly | acinar | 3 | 2 | III |
| 91 | Male | 58 | smoked/smoking | 2019/1/23 | adenocarcinoma | poorly | solid | 3 | 1 | III |
| 92 | Female | 36 | never | 2019/1/31 | adenocarcinoma | poorly | solid | 2 | 2 | III |
| 93 | Female | 32 | never | 2019/2/18 | adenocarcinoma | poorly | papillary | 3 | 2 | III |
| 94 | Male | 59 | smoked/smoking | 2012/10/29 | adenocarcinoma | poorly | solid | 2 | 0 | I |
| 95 | Male | 59 | smoked/smoking | 2015/11/27 | adenocarcinoma | poorly | solid | 2 | 0 | I |
| 96 | Male | 62 | smoked/smoking | 2016/7/14 | adenocarcinoma | poorly | acinar | 2 | 0 | I |
| 97 | Male | 54 | smoked/smoking | 2017/11/14 | adenocarcinoma | poorly | solid | 2 | 0 | I |
