# Supplementary material for: Heterogeneity in intracranial relapses after complete resection of lung adenocarcinoma: Distinct features of brain‐only relapse versus synchronous extracranial relapse
Source: Cancer Med. 2023 Apr 16;12(11):12495–503. doi: 10.1002/cam4.5961 (PMC10278484; doi:10.1002/cam4.5961)
Supplement: Supplementary file 2 — Table S2. [file CAM4-12-12495-s001.docx]

# Supplementary table 2. Radiographic data of intracranial relapse lesions with or without ECM

|  |  | **BM-only group**  **N=50** | | **BM-with group**  **N=47** | | **All BM patients**  **N=97** | ***p* value** |
| --- | --- | --- | --- | --- | --- | --- | --- |
| **BM^*^ number** | 1 | | 32 | | 22 | 54 | 0.064 |
|  | 2-5 | | 11 | | 9 | 20 |  |
|  | >5 | | 7 | | 16 | 23 |  |
| **BM site** | Cerebrum | | 41 | | 32 | 73 | 0.275 |
|  | Cerebellum/Brainstem | | 4 | | 5 | 9 |  |
|  | Both | | 5 | | 10 | 15 |  |
| **BM location** | Right | | 19 | | 15 | 34 | 0.216 |
|  | Reft | | 18 | | 12 | 30 |  |
|  | Both side | | 13 | | 20 | 33 |  |
| **Largest diameter(Mean)** |  | | 1.66cm | | 1.40cm |  | 0.246 |

*BM=Brain Metastases
